# Tembusu-Related Flavivirus in Ducks, Thailand

**DOI:** 10.3201/eid2112.150600

**Published:** 2015-12

**Authors:** Aunyaratana Thontiravong, Patchareeporn Ninvilai, Wikanda Tunterak, Nutthawan Nonthabenjawan, Supassma Chaiyavong, Kingkarn Angkabkingkaew, Chatthapon Mungkundar, Woranuch Phuengpho, Kanisak Oraveerakul, Alongkorn Amonsin

**Affiliations:** Chulalongkorn University, Bangkok, Thailand (A. Thontiravong, P. Ninvilai, W. Tunterak, N. Nonthabenjawan, S. Chaiyavong, K. Oraveerakul, A. Amonsin);; Animal Health and Technical Service Office, Bangkok (P. Ninvilai, K. Angkabkingkaew, C. Mungkundar, W. Phuengpho)

**Keywords:** duck Tembusu virus, ducks, Thailand, flavivirus, viruses, mosquitoes, vector-borne infections

## Abstract

Since 2013, outbreaks of disease caused by duck Tembusu virus (DTMUV) have been observed in layer and broiler duck farms in Thailand. The virus is closely related to Chinese DTMUVs and belongs to the Ntaya group of mosquitoborne flaviviruses. These findings represent the emergence of DTMUV in ducks in Thailand.

In 2010, a severe contagious disease emerged in layer and breeder duck farms in China (*1*). The infected ducks typically exhibited a dramatic reduction in egg production and severe neurologic disorders. The causative agent of this emerging disease was identified as the new duck Tembusu virus (DTMUV), a member of the Ntaya virus group in the genus *Flavivirus* (*1*,*2*). In addition to China, new DTMUV was recently detected among ducks in Malaysia (*3*). In Thailand, a severe contagious disease affecting ducks has newly emerged since 2013. The disease rapidly spread through duck farms in high-density duck-producing areas, causing economic losses for both traditional and agro-industrial duck businesses. This study reports the emergence of DTMUV infection among domestic ducks in Thailand.

## The Study

**Figure 1:**
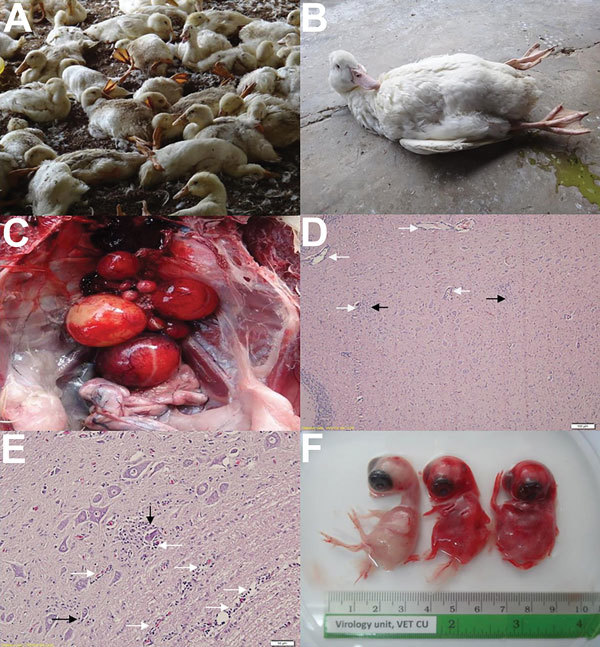
Clinical signs and pathologic lesions of duck Tembusu virus (DTMUV)–infected ducks, Thailand. A, B) Clinical signs; DMTUV-infected ducks showed neurologic signs, including inability to stand, ataxia, and paralysis. C) Gross lesion; severe hemorrhage and regression of ovarian follicles. D, E) Histopathologic lesion; moderate multifocal gliosis (black arrow) and perivascular cuffing (white arrow) in cerebellum (D) and spinal cord (E). Scale bars indicate 100 μm (D) and 50 μm (E). F) Chicken embryos infected with DTMUV strain DK/TH/CU-1. Normal embryo is shown at left; infected embryos at right died 3–5 days after inoculation, with severe cutaneous hemorrhage.

Since 2013, several layer and broiler duck farms located in high-density duck-producing areas of Thailand have had an emerging, contagious disease characterized by severe neurologic dysfunction and dramatically decreased egg production among domestic ducks. Outbreaks have been reported on farms in the northeastern (Nakhon Ratchasima), eastern (Prachinburi and Chonburi), and central (Suphanburi) provinces of Thailand. At least 7 duck farms were affected, and outbreaks occurred throughout the year (August 2013–September 2014). However, the disease occurred more frequently during the rainy season (July–December). We estimated the mean prevalence of the outbreaks at 17.19% (Technical Appendix Figure 1, panels A, B). Clinical signs were usually observable in broiler ducks >3 weeks of age and in layer ducks during their production period. Infected ducks typically exhibited neurologic signs, including ataxia, reluctance to walk, and progressive paralysis (Figure 1, panels A, B). A remarkable drop in egg production was usually observed among layer ducks. The main pathologic changes were ovaritis, ovarian hemorrhage, and ovarian atrophy (Figure 1, panel C). Splenic enlargement was observed in some ducks. Histopathologic analysis showed moderate multifocal gliosis and perivascular cuffing in the brain (cerebellum) and spinal cord of most sick ducks (Figure 1, panels D, E). Rates of illness and death ranged from 20% to 50% and 10% to 30%, respectively, correlating positively with secondary bacterial infection.

We identified 22 DTMUVs through reverse transcription PCR using E gene–specific primers (*1*) (Technical Appendix Table 1). One virus (DK/TH/CU-1) was inoculated into embryonated chicken eggs. The embryos died within 3–5 days after inoculation, with severe cutaneous hemorrhages (Figure 1, panel F). The allantoic fluid tested negative through hemagglutination test and PCR for common duck viruses, including avian influenza virus, Newcastle disease virus and duck herpesvirus 1. In addition, 5 representative viruses (DK/TH/CU-2, DK/TH/CU-3, DK/TH/CU-4, DK/TH/CU-5, DK/TH/CU-6) from duck farms located in the northeastern (3 farms) and the eastern (2 farms) provinces were selected for partial E gene sequencing (Table). The nucleotide sequences of the Thai DMTUVs used in this study were submitted to GenBank under accession nos. KR061333–KR061338.

**Table:** Detailed description of DTMUVs characterized in study of DTMUV in ducks, Thailand*

Virus name	Study designation	Time of collection	Duck age	Duck type	Location in Thailand	Genome sequencing	GenBank accession no.
DTMUV strain DK/TH/CU-1	DK/TH/CU-1	2013 Nov	39 d	Broiler	Nakhon Ratchasima	WG	KR061333
DTMUV strain DK/TH/CU-2	DK/TH/CU-2	2014 Aug	38 wk	Layer	Chonburi	Partial E	KR061334
DTMUV strain DK/TH/CU-3	DK/TH/CU-3	2014 Aug	35 d	Broiler	Nakhon Ratchasima	Partial E	KR061335
DTMUV strain DK/TH/CU-4	DK/TH/CU-4	2014 Aug	42 d	Broiler	Nakhon Ratchasima	Partial E	KR061336
DTMUV strain DK/TH/CU-5	DK/TH/CU-5	2013 Sep	24 d	Broiler	Nakhon Ratchasima	Partial E	KR061337
DTMUV strain DK/TH/CU-6	DK/TH/CU-6	2013 Oct	35 d	Broiler	Prachinburi	Partial E	KR061338

*All samples were pooled organs (i.e., brain, spinal cord, spleen, lung, kidney, proventiculus, and intestine). DTMUV, duck Tembusu virus; partial E, partial E gene sequence; WG, whole-genome.

**Figure 2:**
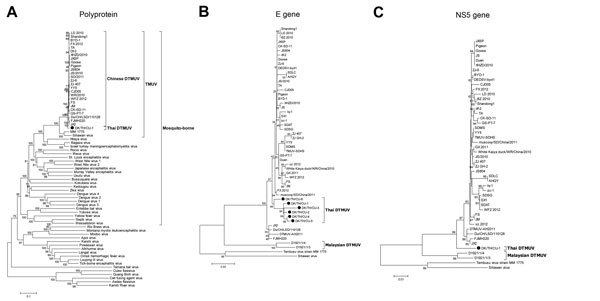
Phylogenetic analysis of the nucleotide sequences of polyprotein gene (10,278 bp) (A), partial envelope gene (361 bp) (B), and partial nonstructural 5 gene (900 bp) (C) of duck Tembusu viruses (DTMUVs) from ducks in Thailand and selected reference strains of flaviviruses. The nucleotide sequences were aligned by using Muscle version 3.6 (*4*). The phylogenetic trees were constructed in MEGA version 6.0 by using the neighbor-joining algorithm with the Kimura-2 parameter model applied to 1,000 replications of bootstrap (*5*). Circle indicates Thai DTMUVs. Similar results were observed when applying the maximum-likelihood algorithm (Technical Appendix Figure 2). Scale bars indicate nucleotide substitutions per site.

To characterize Thai DTMUV, DK/TH/CU-1 was subjected to whole-genome sequencing. The whole-genome length of DK/TH/CU-1 is 10,278 nt, encoding 3,426 aa. BLAST analysis (http://www.ncbi.nlm.nih.gov/blast) showed that the polyprotein gene sequences of DK/TH/CU-1 shared very high identity (98.3%) with GX2013E, a Chinese DTMUV strain isolated in 2013. Phylogenetic analysis of the polyprotein gene sequence using the neighbor-joining and maximum-likelihood algorithms showed that DK/TH/CU-1 is grouped into the major cluster with mosquito-borne flaviviruses (65.2%–77% nt identity with viruses in the Ntaya group) and is most closely related to Chinese DTMUVs (97.3%−98.3% nt identity). DK/TH/CU-1 shared only 90.3% and 89.4% nt identity with MM1775 strain and Sitiawan virus, which are Tembusu viruses isolated from mosquitos and chickens, respectively (Figure 2, panel A; Technical Appendix Table 2).

Analysis of the partial E gene sequences of the 5 Thai DTMUVs (DK/TH/CU-2 to 6) showed that the viruses are grouped with DK/TH/CU-1 and Chinese DTMUVs (Figure 2, panel B). The partial E gene sequences of Thai DTMUVs shared 97.5%−99.7% and 96.7%−98.9% nt identity with each other and with the Chinese DTMUVs, respectively. However, the nucleotide identities were lower (88.6%–90.6%) than Malaysian DTMUVs. The E gene sequence of a DK/TH/CU-1 shares only 89.1% and 90.9% nt identity with TMUV strains isolated in 2002 from mosquitos and healthy ducks in Thailand, respectively (*6*). Analysis of partial NS5 gene showed similar findings with those of polyprotein and E genes (Figure 2, panel C). DK/TH/CU-1 shared 96.4%–98.1% and 92.7%–93% nt identity with Chinese DTMUVs and Malaysian DTMUVs, respectively.

## Conclusions

Since 2013, outbreaks of a severe contagious disease among domestic ducks have been occurring and spreading in the high-density duck-producing areas of Thailand, causing substantial economic losses in the agricultural sector. On the basis of pathologic examinations, virus isolation, virus identification and genetic characterization, we found an association with the new DTMUV.

Despite lack of the experimental pathogenicity testing of the virus isolates, our observations on clinical signs and pathologic findings were consistent with previously reported findings of DTMUV infections in China and Malaysia (*1*,*3*). Therefore, the isolated DTMUVs can be considered as the causative agent. Because DTMUV is a mosquito-borne flavivirus, it can be transmitted to ducks from mosquitos. Our data indicated that the disease caused by DTMUV occurred most frequently during rainy season, when mosquito activity in Thailand is highest. A previous study detected TMUV in *Culex* mosquitos in Thailand in 2002. The *Culex* mosquito has also proven to be a vector for transmitting TMUV to chickens (*6*). DTMUV transmission through the fecal–oral route also has been reported (*2*,*7*,*8*). However, the pathogenicity and transmission routes of Thai DTMUV were not determined in this study. Further studies on the Thai DTMUVs should be conducted.

Genetic analyses of polyprotein sequences of the Thai DTMUVs showed higher nucleotide identity with DTMUVs reported from China (97.9%) than with those reported from Malaysia (90.3%), indicating that Chinese DTMUVs are possible ancestors of Thai DTMUVs. Phylogenetic analyses based on polyprotein, E gene and NS5 gene using 2 algorithms (neighbor-joining and maximum-likelihood) have displayed similar results that the Thai isolates were grouped with the Chinese DTMUV with high bootstraps value. The Malaysian DTMUVs were grouped into a subcluster apart from Thai and Chinese DTMUV. Although TMUV strains were isolated from Thai mosquitos and healthy ducks in 2002, the nucleotide sequences of those viruses were less similar to Thai DTMUVs than those of Chinese DTMUVs. Nevertheless, the source of the novel DTMUV emergence in Thailand remains unknown and requires further investigation.

As a member of the *Flavivirus* genus, DTMUV has a high potential to become a zoonotic pathogen that threatens public health. Thus far, DTMUV has not been reported to cause illness in humans. However, DTMUV-specific antibodies and DTMUV RNA were detected in duck farm workers in China (*9*). Therefore, a novel DTMUV that can cause disease in humans possibly could emerge. Previous studies have reported that DTMUV can infect a wide variety of avian species, including geese, chickens, pigeons, and house sparrows, indicating the continued expansion of its host range (*7*,*10*–*12*). Thus, the continued monitoring of DTMUV in animals and humans is essential to preventing economic losses in animal production as well as zoonotic potential in humans. In summary, our data collectively demonstrate that a newly emerged, contagious disease among ducks in Thailand is caused by DTMUV. Our findings highlight the necessity of systemic surveillance of DTMUVs in animals and in humans for early detection and prevention.

Technical Appendix Detailed methods for study of duck Tembusu virus in ducks, Thailand.
