# Supplementary material for: Tembusu-Related Flavivirus in Ducks, Thailand
Source: Emerg Infect Dis. 2015 Dec;21(12):2164–7. doi: 10.3201/eid2112.150600 (PMC4672441; doi:10.3201/eid2112.150600)
Supplement: Technical Appendix — Detailed methods for study of duck Tembusu virus in ducks, Thailand. [file 15-0600-Techapp-s1.pdf]

Article DOI: http://dx.doi.org/10.3201/eid2112.150600

Tembusu-Related Flavivirus in Ducks, Thailand

Technical Appendix

Methods

Outbreak Investigations

During August 2013–September 2014, we investigated outbreaks of a contagious duck disease among ducks characterized by severe neurologic dysfunction and dramatic decreases in egg production in layer and broiler duck farms in Thailand. Epidemiologic information, clinical observations, postmortem examinations, samples collection, and laboratory testing were recorded and analyzed to determine the etiology of the outbreaks.

Virus Isolation and Identification

Visceral organ samples were collected from affected ducks, including brain, spinal cord, spleen, lung, kidney, proventiculus, and intestine. Each sample was homogenized in sterile phosphate-buffered saline at a 10% suspension (w/v), centrifuged at 3,000 × g for 15 min, then filtered through 0.2-µm filters. The filtered suspensions were inoculated into the allantoic cavities of 9-day-old embryonated chicken eggs. The allantoic fluids and tissue suspensions were then examined for the presence of duck Tembusu virus (DTMUV) by reverse transcription PCR (RT-PCR) by using E gene–specific primers (*1*).

The samples were also tested for avian influenza virus (*2*), Newcastle disease virus (*3*,*4*), and duck herpesvirus (*5*) to rule out other common viruses that can cause similar symptoms. The tissue suspensions and virus isolates were also tested by hemagglutination tests against 1% chicken erythrocytes at 25°C, pH 7.4 to exclude avian hemagglutinating viruses, including avian influenza virus and Newcastle disease virus.

Whole-Genome Sequencing and Phylogenetic Analysis of Thai DTMUV

In this study, 1 DTMUV isolate from Thailand (DK/TH/CU-1) was selected and subjected to whole-genome sequencing. Additionally, 5 other isolates (DK/TH/CU-2,

DK/TH/CU-3, DK/TH/CU-4, DK/TH/CU-5, and DK/TH/CU-6) were subjected to partial E gene sequencing. Viral RNAs were extracted from allantoic fluid by using NucleoSpin Extract Viral RNA Kit (Macherey-Nagel, Düren, Germany) in accordance with the manufacturer's instructions. RT-PCR was performed in a single-step reaction by using the AccessQuick RT-PCR System (Promega, Madison, WI, USA). Primers were designed on the basis of the complete genome sequences of DTMUV available in GenBank. The oligonucleotide primer sequences are shown in online Technical Appendix Table 1. The amplicons were then subjected to DNA sequencing (1st Base Laboratories Sdn Bhd, Malaysia). The validated nucleotide sequences were assembled by using SeqMan software v.5.03 (DNASTAR Inc., Madison, Wisconsin, USA). These nucleotide sequences of the Thai DMTUV were submitted to GenBank under accession nos. KR061333–8.

Phylogenetic analysis was performed by comparing the nucleotide sequences of polyprotein, E gene and NS genes of the Thai DMTUVs with those of other reference flaviviruses available in GenBank. The nucleotide sequences were aligned by using Muscle v.3.6 (*7*). The phylogenetic trees were constructed in MEGA v.6.0 by using the neighbor-joining and maximum-likelihood algorithms (*8*). To determine nucleotide identities, the nucleotide sequences of Thai, Malaysian, and Chinese DTMUVs were aligned and compared in MegAlign software v.5.03 (DNASTAR Inc.) with other reference flaviviruses.

References

1. Su J, Li S, Hu X, Yu X, Wang Y, Liu P, et al. Duck egg-drop syndrome caused by BYD virus, a new Tembusu-related flavivirus. PLoS ONE. 2011;6:e18106. PubMed http://dx.doi.org/10.1371/journal.pone.0018106

2. Spackman E, Senne DA, Myers TJ, Bulaga LL, Garber LP, Perdue ML, et al. Development of a real-time reverse transcriptase PCR assay for type A influenza virus and the avian H5 and H7 hemagglutinin subtypes. J Clin Microbiol. 2002;40:3256–60. PubMed http://dx.doi.org/10.1128/JCM.40.9.3256-3260.2002

3. Ke GM, Yu SW, Ho CH, Chu PY, Ke LY, Lin KH, et al. Characterization of newly emerging Newcastle disease viruses isolated during 2002–2008 in Taiwan. Virus Res. 2010;147:247–57. PubMed http://dx.doi.org/10.1016/j.virusres.2009.11.008

4. Liu H, Wang Z, Wu Y, Zheng D, Sun C, Bi D, et al. Molecular epidemiological analysis of Newcastle disease virus isolated in China in 2005. J Virol Methods. 2007;140:206–11. PubMed http://dx.doi.org/10.1016/j.jviromet.2006.10.012

5. Li H, Liu S, Kong X. Characterization of the genes encoding UL24, TK and gH proteins from duck enteritis virus (DEV): a proof for the classification of DEV. Virus Genes. 2006;33:221–7. PubMed http://dx.doi.org/10.1007/s11262-005-0060-6

9. Huang X, Han K, Zhao D, Liu Y, Zhang J, Niu H, et al. Identification and molecular characterization of a novel flavivirus isolated from geese in China. Res Vet Sci. 2013;94:774–80. PubMed http://dx.doi.org/10.1016/j.rvsc.2012.11.014

7. Edgar RC. MUSCLE: a multiple sequence alignment method with reduced time and space complexity. BMC Bioinformatics. 2004;5:113. PubMed http://dx.doi.org/10.1186/1471-2105-5-113

8. Tamura K, Peterson D, Peterson N, Stecher G, Nei M, Kumar S. MEGA5: molecular evolutionary genetics analysis using maximum likelihood, evolutionary distance, and maximum parsimony methods. Mol Biol Evol. 2011;28:2731–9. PubMed http://dx.doi.org/10.1093/molbev/msr121

Technical Appendix Table 1. Primers used for whole-genome sequencing of a Thai duck Tembusu virus

Primer name	Sequence (5′→3′)	Reference
P1f	AGAAGTTCRYCTGTGTGA	(*6*)
DF_R638	CAGCAGTCTATGTCTTCAGG	This study
DF_F441	CGATAGTTGCTGGGCTGAAGC	This study
DF_R1115	GCAGTAAGATCTCACAACCGC	This study
DF_F954	GCTTCAGCTGTCTGGGGATGC	This study
DF_R1650	CAATGACTCTTTGTTTTGCCACG	This study
DF_F1480	CTACACYGCTGAGATGGAGG	This study
DF_R2463	GCCAAGTCGATTGAGCACCCC	This study
DF_F2353	GGCACTGCTATTGTGGATGGG	This study
DF_R3339	GGTGGGGTGGTGCAAGACC	This study
DF_F3302	GGAACAACTGTCACAGTAACG	This study
DF_R4694	GCATGACTCCCACTCCAGCC	This study
DF_F4406	GCATCACAGAGATTTGATGTGG	This study
DF_R5162	CCTGAACCTGGATGTAGGTCC	This study
DF_F4874	GCAAGTCATCGTCGTGCAACC	This study
DF_R5582	GCTCTTCAATGTCTGTTATTGGC	This study
DF_F5399	GCTCACACCTCAGCGAGTGC	This study
DF_R6249	GGTCATTGTAACTTATCCCAGC	This study
DF_F5928	CCAGTTCCTATAACATCAGCC	This study
DF_R6678	GCTCCAAGACCTGTCTTCCC	This study
DF_F6494	CGCTCACAGAATGACAGAATCC	This study
DF_R7348	GGAACATCTGTAGCCACTATGC	This study
DF_F6807	GAACCAGAGAGACAGAGATCGC	This study
DF_R8158	CCCTAGCTAGCCATTCCTCGG	This study
DF_F7940	GCAGGTTCAGGAAGTGAGAGG	This study
DF_R8536	GGATTGTCTTGGTCATAATGCC	This study
DF_F8383	GGATGCACAAAACCAACCGC	This study
DF_R9215	GGCCGAGATGTCACGCAGC	This study
DF_F8084	GCTGTGTGACATAGGTGAAGC	This study
DF_R9449	CCACTTCCCCTCTGGTCTTCC	This study
DF_F9274	GGGACACTAGAATAACCAAGGC	This study
DF_R10485	CCAACATCCGGTGGCAGGG	This study

Technical Appendix Table 2. Nucleotide identities of the polyprotein gene sequences of a Thai DTMUV (DK/TH/CU-1) with reference viruses of Ntaya virus group

	DK/TH/CU-1	WFZ_2012	CJD05	xz_2012	YY5	goose	pigeon	DEDSV-byd1	BYD-1	MM1775	STWV	NTAV	BAGV	ITV	ROCV	ILHV	ZIKV
DK/TH/CU-1	100	97.7	97.6	97.7	97.9	97.8	97.9	97.9	97.9	90.3	89.4	76.9	76.7	77	69.4	68.8	65.2
WFZ_2012			99.1	99.2	99.4	99.3	99.3	99.4	99.4	90.6	89.4	76.9	76.5	77	69.4	68.6	65.2
CJD05				99.1	99.3	99.2	99.3	99.4	99.4	90.6	89.3	76.9	76.5	76.8	69.3	68.6	65.3
xz_2012					99.4	99.3	99.3	99.4	99.4	90.7	89.5	77	76.5	76.8	69.3	68.7	65.4
YY5						99.5	99.6	99.6	99.6	90.7	89.5	77	76.6	76.9	69.4	68.7	65.3
goose							99.9	99.6	99.6	90.6	89.5	77	76.5	76.8	69.4	68.7	65.3
pigeon								99.6	99.6	90.7	89.5	77	76.5	76.9	69.4	68.7	65.3
DEDSV-byd1									100	90.7	89.5	77	76.6	76.9	69.4	68.7	65.3
BYD-1										90.7	89.5	77	76.6	76.9	69.4	68.7	65.3
MM1775											94	76.5	76.6	76.9	69.3	68.6	65.1
STWV												76.5	76.5	76.8	69.2	68.4	65.1
NTAV													79.9	80.2	69	68.5	65.2
BAGV														94.9	69.3	69.1	65.2
ITV															69.6	69.1	65.2
ROCV																74.9	65.4
ILHV																	65.4
ZIKV																	100

*BAGV, Bagaza virus; DK/TH/CU-1, Thai duck tembusu virus; ILHV, Ilheus virus; ITV, Israel turkey meningoencephalomyelitis virus; WFZ_2012, CJD05, xz_2012, YY5, goose, pigeon, DEDSV-byd1, BYD-1, Chinese duck tembusu viruses; MM1775, Tembusu virus strain MM1775; NTAV, Ntaya virus; ROCV, Rocio virus; STWV, Sitiawan virus; ZIKV, Zika virus.

A

B

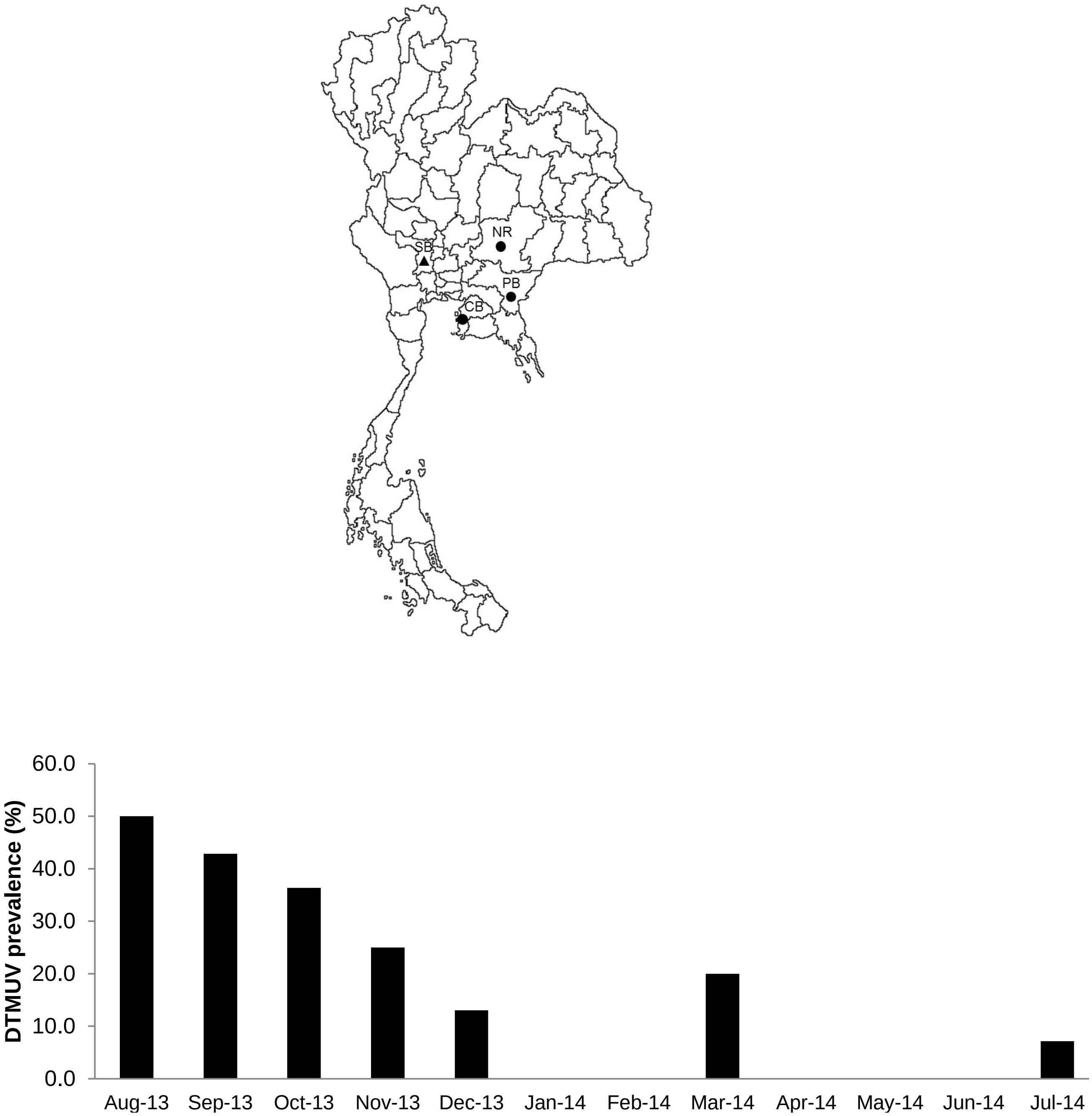

Technical Appendix Figure 1. Details of DTMUV outbreaks in Thailand during 2013–2014. A) Locations of DTMUV outbreaks in Thailand. Circles represent provinces where the DMTUV isolated. NR, Nakhon Ratchasima; PC, Prachinburi; CB, Chonburi; SB, Suphanburi. B) Monthly prevalence of DTMUV in farmed ducks, Thailand, 2013–2014. DTMUV, duck Tembusu virus.

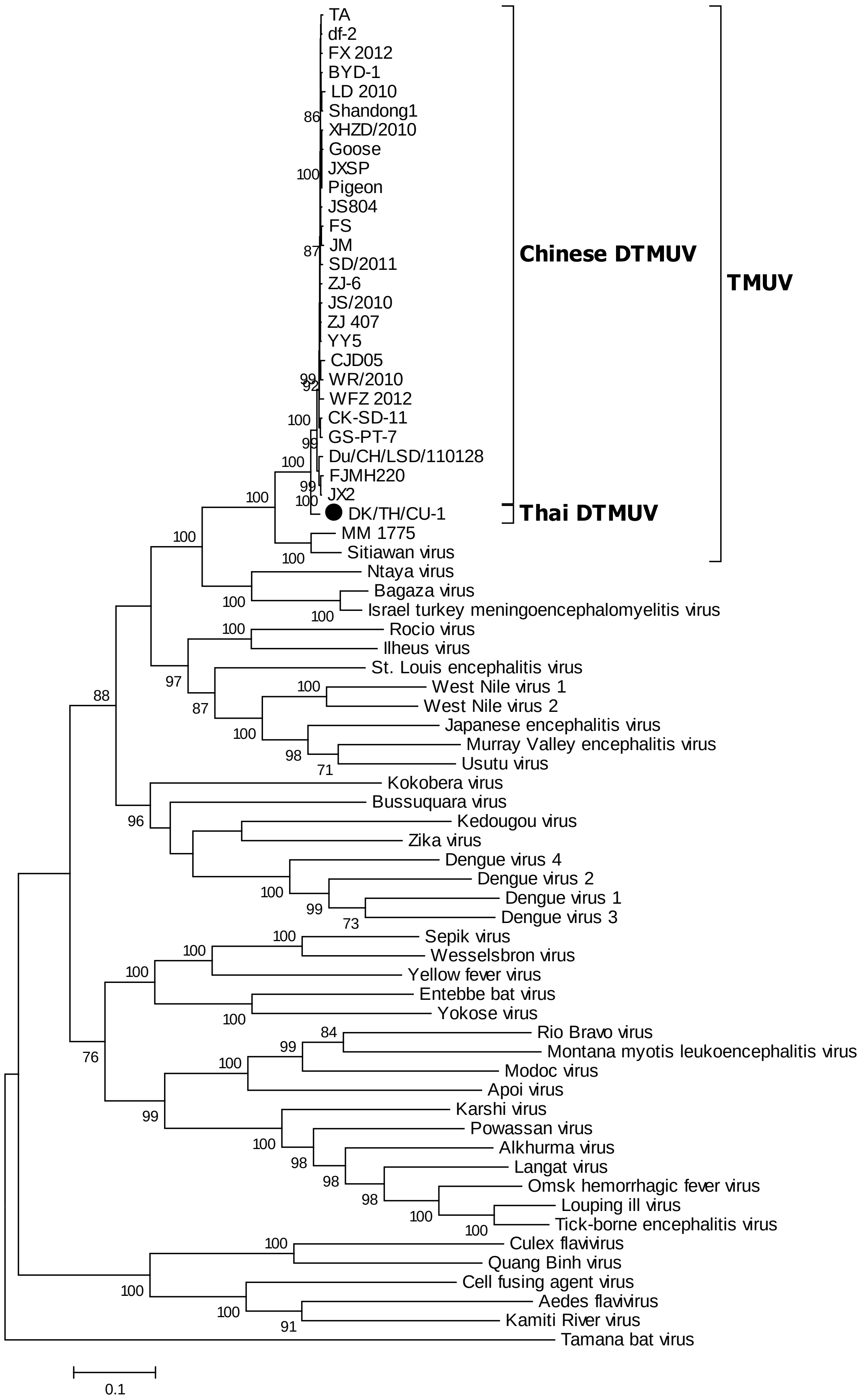

TA
df-2
FX 2012
BYD-1
LD 2010
Shandong1
XHZD/2010
Goose
JXSP
Pigeon
JS804
FS
JM
SD/2011
ZJ-6
JS/2010
ZJ 407
YY5
CJD05
WR/2010
WFZ 2012
CK-SD-11
GS-PT-7
Du/CH/LSD/110128
FJMH220
JX2
DK/TH/CU-1
MM 1775
Sitiawan virus
Ntaya virus
Bagaza virus
Israel turkey meningoencephalomyelitis virus
Rocio virus
Ilheus virus
St. Louis encephalitis virus
West Nile virus 1
West Nile virus 2
Japanese encephalitis virus
Murray Valley encephalitis virus
Usutu virus
Kokobera virus
Bussuquara virus
Kedougou virus
Zika virus
Dengue virus 4
Dengue virus 2
Dengue virus 1
Dengue virus 3
Sepik virus
Wesselsbron virus
Yellow fever virus
Entebbe bat virus
Yokose virus
Rio Bravo virus
Montana myotis leukoencephalitis virus
Modoc virus
Apoi virus
Karshi virus
Powassan virus
Alkhurma virus
Langat virus
Omsk hemorrhagic fever virus
Louping ill virus
Tick-borne encephalitis virus
Culex flavivirus
Quang Binh virus
Cell fusing agent virus
Aedes flavivirus
Kamiti River virus
Tamana bat virus
Chinese DTMUV
Thai DTMUV
TMUV
0.1

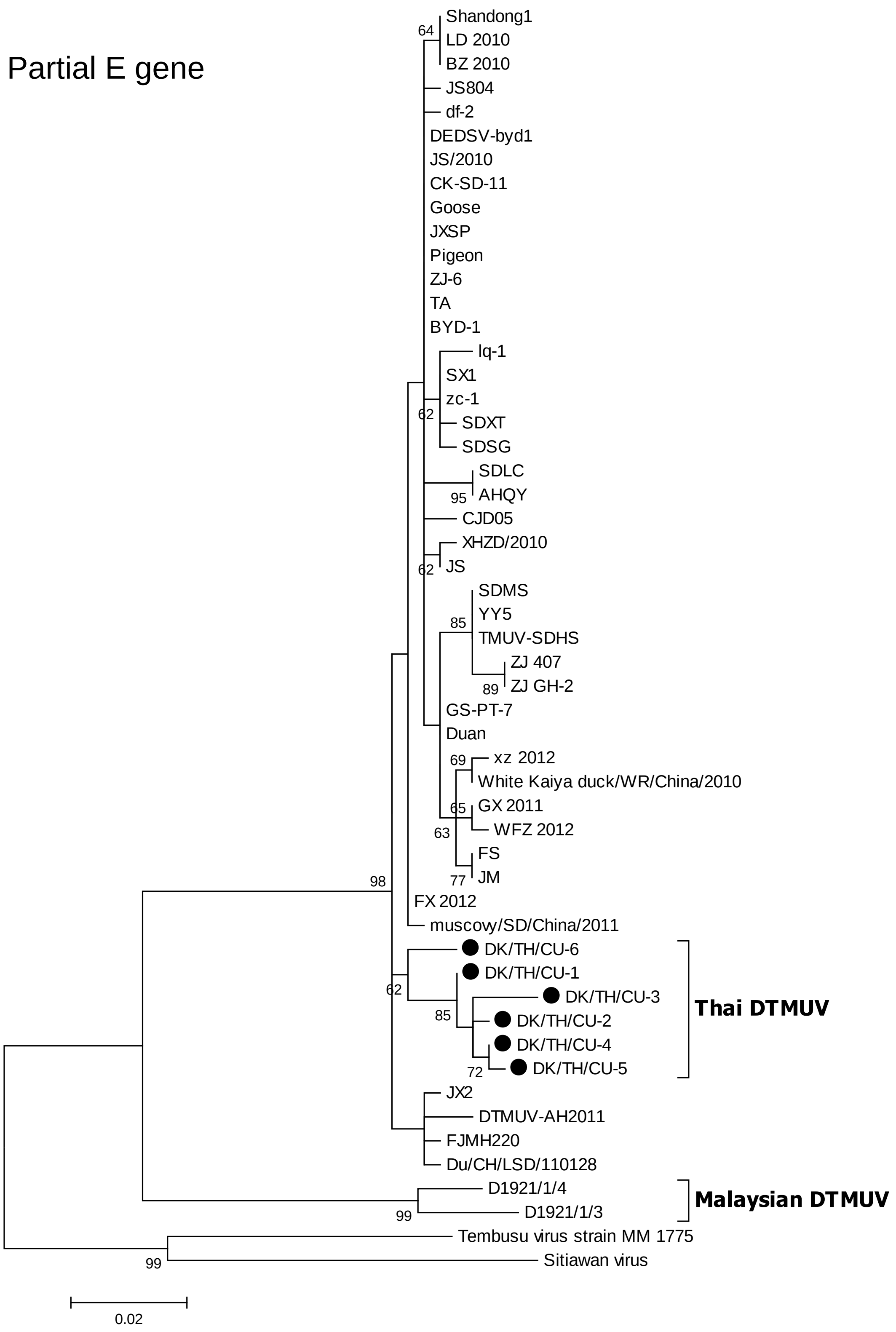

Partial E gene
Shandong1
LD 2010
BZ 2010
JS804
df-2
DEDSV-byd1
JS/2010
CK-SD-11
Goose
JXSP
Pigeon
ZJ-6
TA
BYD-1
lq-1
SX1
zc-1
SDXT
SDSG
SDLC
AHQY
CJD05
XHZD/2010
JS
SDMS
YY5
TMUV-SDHS
ZJ 407
ZJ GH-2
GS-PT-7
Duan
xz 2012
White Kaiya duck/WR/China/2010
GX 2011
WFZ 2012
FS
JM
FX 2012
muscovy/SD/China/2011
DK/TH/CU-6
DK/TH/CU-1
DK/TH/CU-3
DK/TH/CU-2
DK/TH/CU-4
DK/TH/CU-5
Thai DTMUV
JX2
DTMUV-AH2011
FJMH220
Du/CH/LSD/110128
D1921/1/4
D1921/1/3
Malaysian DTMUV
Tembusu virus strain MM 1775
Sitiawan virus
0.02

NS5 gene

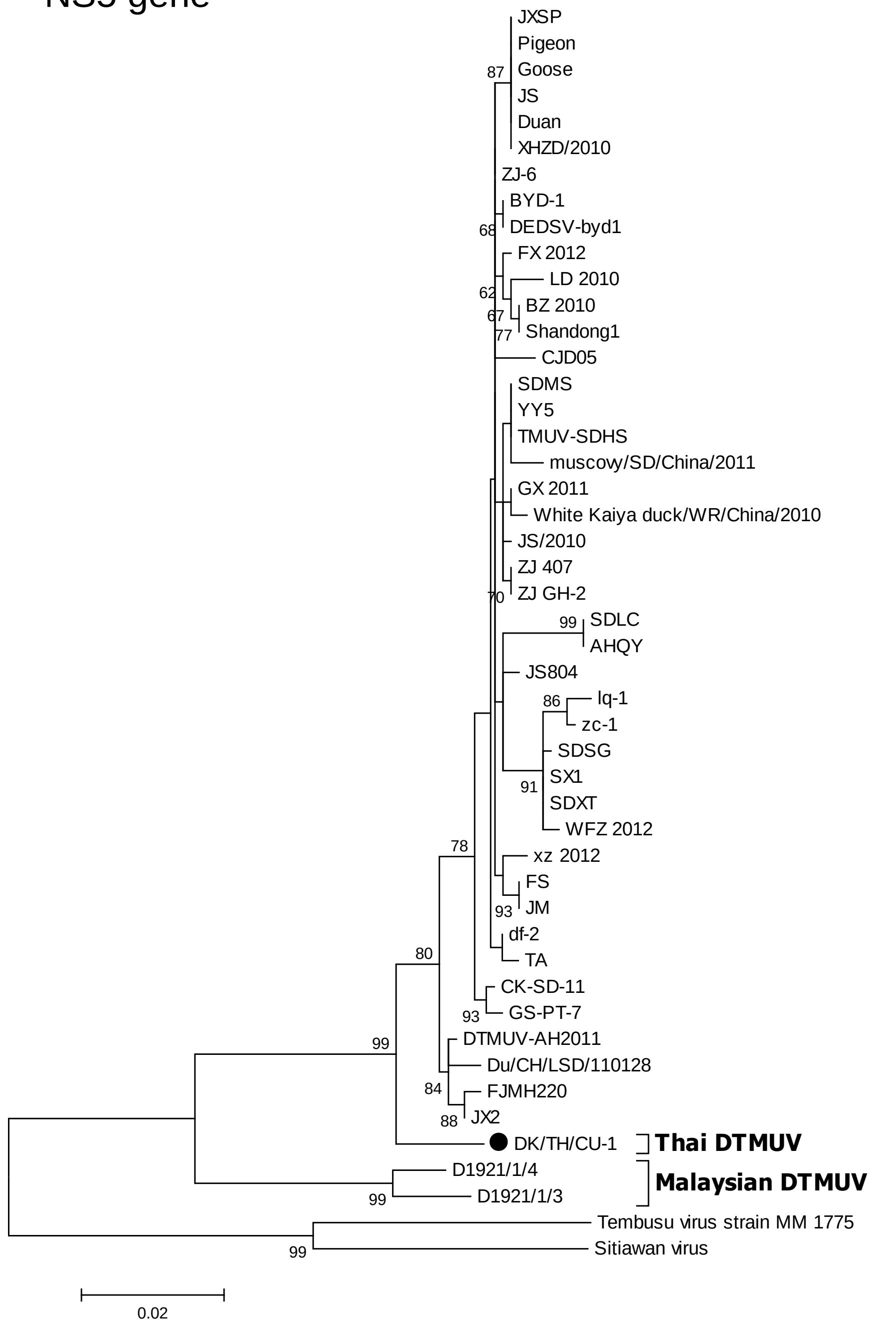

Technical Appendix Figure 2. Phylogenetic analysis of the nucleotide sequences of polyprotein gene (10,278 bp), partial E gene (361 bp), and partial NS gene (900 bp) of Thai DTMUVs and selected reference strains of flaviviruses. The nucleotide sequences were aligned by using Muscle v.3.6 (*7*). The phylogenetic trees were constructed in MEGA v.6.0 by using the maximum-likelihood algorithm with the Hasegawa-Kishino-Yano (HKY) model applied to 500 replications of bootstrap (*8*). Circle indicates Thai DTMUVs.
